# Predictive value of pretreatment circulating inflammatory response markers in the neoadjuvant treatment of breast cancer: meta-analysis

**DOI:** 10.1093/bjs/znae132

**Published:** 2024-05-27

**Authors:** Gavin P Dowling, Gordon R Daly, Aisling Hegarty, Sandra Hembrecht, Aisling Bracken, Sinead Toomey, Bryan T Hennessy, Arnold D K Hill

**Affiliations:** Department of Surgery, Royal College of Surgeons in Ireland (RCSI) University of Medicine and Health Sciences, Dublin, Ireland; Medical Oncology Lab, Department of Molecular Medicine, Royal College of Surgeons in Ireland (RCSI) University of Medicine and Health Sciences, Dublin, Ireland; Department of Surgery, Beaumont Hospital, Dublin, Ireland; Department of Surgery, Royal College of Surgeons in Ireland (RCSI) University of Medicine and Health Sciences, Dublin, Ireland; Department of Surgery, Beaumont Hospital, Dublin, Ireland; Department of Surgery, Royal College of Surgeons in Ireland (RCSI) University of Medicine and Health Sciences, Dublin, Ireland; Department of Surgery, Beaumont Hospital, Dublin, Ireland; Department of Surgery, Royal College of Surgeons in Ireland (RCSI) University of Medicine and Health Sciences, Dublin, Ireland; Department of Surgery, Beaumont Hospital, Dublin, Ireland; Department of Surgery, Royal College of Surgeons in Ireland (RCSI) University of Medicine and Health Sciences, Dublin, Ireland; Medical Oncology Lab, Department of Molecular Medicine, Royal College of Surgeons in Ireland (RCSI) University of Medicine and Health Sciences, Dublin, Ireland; Medical Oncology Lab, Department of Molecular Medicine, Royal College of Surgeons in Ireland (RCSI) University of Medicine and Health Sciences, Dublin, Ireland; Department of Surgery, Royal College of Surgeons in Ireland (RCSI) University of Medicine and Health Sciences, Dublin, Ireland; Department of Surgery, Beaumont Hospital, Dublin, Ireland

## Abstract

**Background:**

Systemic inflammatory response markers have been found to have a prognostic role in several cancers, but their value in predicting the response to neoadjuvant chemotherapy in breast cancer is uncertain. A systematic review and meta-analysis of the literature was carried out to investigate this.

**Methods:**

A systematic search of electronic databases was conducted to identify studies that explored the predictive value of circulating systemic inflammatory response markers in patients with breast cancer before commencing neoadjuvant therapy. A meta-analysis was undertaken for each inflammatory marker where three or more studies reported pCR rates in relation to the inflammatory marker. Outcome data are reported as ORs and 95% confidence intervals.

**Results:**

A total of 49 studies were included, of which 42 were suitable for meta-analysis. A lower pretreatment neutrophil-to-lymphocyte ratio was associated with an increased pCR rate (pooled OR 1.66 (95% c.i. 1.32 to 2.09); *P* < 0.001). A lower white cell count (OR 1.96 (95% c.i. 1.29 to 2.97); *P* = 0.002) and a lower monocyte count (OR 3.20 (95% c.i. 1.71 to 5.97); *P* < 0.001) were also associated with a pCR. A higher lymphocyte count was associated with an increased pCR rate (OR 0.44 (95% c.i. 0.30 to 0.64); *P* < 0.001).

**Conclusion:**

The present study found the pretreatment neutrophil-to-lymphocyte ratio, white cell count, lymphocyte count, and monocyte count of value in the prediction of a pCR in the neoadjuvant treatment of breast cancer. Further research is required to determine their value in specific breast cancer subtypes and to establish optimal cut-off values, before their adoption in clinical practice.

## Teaser

The present study found the pretreatment neutrophil-lymphocyte-ratio, white cell count, lymphocyte count, and monocyte count of value in the prediction of a pCR in the neoadjuvant treatment of breast cancer.

## Introduction

There is increasing evidence over the last few decades to support the use of neoadjuvant systemic therapy in the management of breast cancer. The benefits of neoadjuvant breast cancer treatment are well established, including the potential to down-stage disease and reduce the extent of breast and axillary surgery. Additional benefits include the potential to personalize adjuvant therapy options based on pathological response and to allow for assessment of tumour response to systemic therapy *in vivo*. A pCR is defined as the absence of residual invasive cancer in the resected breast specimen and all sampled regional lymph nodes after the completion of neoadjuvant systemic therapy (ypT0/Tis ypN0)^1^. Favourable long-term oncological outcomes have been associated with a pCR at surgery, particularly in patients with triple-negative breast cancer (TNBC) and human epidermal growth factor receptor 2 (HER2)-positive tumours^2,3^. Therefore, it is necessary to identify a method to accurately predict which patients are likely to achieve a pCR and are thus optimal candidates for neoadjuvant systemic therapy.

It is well understood that the host systemic inflammatory response plays a role in tumour development and progression^4,5^. Serum inflammatory markers have been proposed as prognostic markers in various cancer types, including breast cancer^6,7^. Individual studies have suggested that these markers of systemic inflammation may be of value in predicting the response of breast tumours to neoadjuvant therapy^8,9^. As the majority of these markers are included in routine pretreatment blood tests, this may offer an inexpensive and easily attainable method of optimizing patient treatment. These inflammatory markers include components of the white cell count (WCC), such as neutrophils, lymphocytes, and platelets, and acute-phase proteins, such as C-reactive protein (CRP) and albumin. Scores derived from these markers include the neutrophil-to-lymphocyte ratio (NLR), the platelet-to-lymphocyte ratio (PLR), the lymphocyte-to-monocyte ratio (LMR), the systemic immune-inflammation index (SII), and the systemic inflammation response index (SIRI). These scores have been shown to have prognostic value in several forms of cancer at various stages^10–12^.

While there is evidence that preoperative levels of these markers have a prognostic role, there is little information on their value in predicting breast cancer response to neoadjuvant chemotherapy before commencing treatment. Therefore, a systematic review and meta-analysis of the literature was conducted to assess the value of pretreatment circulating inflammatory markers for predicting a pCR after neoadjuvant therapy.

## Methods

A systematic review and meta-analysis was performed in accordance with Preferred Reporting Items for Systematic reviews and Meta-Analyses (PRISMA)^13^ and Meta-analysis Of Observational Studies in Epidemiology (MOOSE)^14^ guidelines. All authors contributed to formulating the study protocol, which was registered in PROSPERO, the international prospective register of systematic reviews (CRD42023441777). A total of three researchers designed the literature search, retrieved relevant abstracts and full manuscripts, appraised selected studies, and analysed relevant data.

### Inclusion and exclusion criteria

Studies eligible for inclusion were available full English language papers investigating pCR rates in relation to one or more pretreatment serum inflammatory markers or a related score. To be included in the review, studies had to only include patients with breast cancer or, when several cancers were being studied, have identifiable outcomes for the breast cancer subcohort. Studies also had to meet the following inclusion criteria: participants received neoadjuvant treatment for breast cancer; pretreatment serum inflammatory marker or related score was documented; cut-off value for pretreatment serum inflammatory marker or related score was documented; and pCR status after neoadjuvant treatment was recorded. Studies were excluded from the analysis if: the participants did not receive neoadjuvant treatment for breast cancer; and if there was incomplete recording of pretreatment inflammatory marker and pCR status.

### Study selection

A systematic review of PubMed, Embase, the Cochrane Library, and Scopus was performed. Any study published before 1 June 2023 was eligible for inclusion. Combinations of Medical Subject Heading terms and keywords, as well as Boolean operators ‘and’ and ‘or’ were used. Search terms used were ‘breast neoplasm’, ‘neoadjuvant therapy’, ‘white cell count’, ‘neutrophils’, ‘lymphocytes’, ‘monocytes’, ‘platelets’, ‘neutrophil-lymphocyte ratio’, ‘platelet-lymphocyte ratio’, ‘lymphocyte-monocyte ratio’, ‘albumin’, and ‘CRP’. Additionally, the reference lists of the included manuscripts were searched for further eligible studies. Titles and abstracts were initially reviewed for suitability and duplicates were manually removed. Full texts of the remaining articles were then reviewed. Assessment of selected articles regarding inclusion criteria was undertaken independently by two authors and subsequently verified by senior authors. Where two papers by the same author appeared to use the same cohort, the more recent paper with the larger cohort and longer follow-up was retained.

### Data collection and assessment of quality

A pair of investigators (G.P.D. and G.R.D.) independently extracted the following data: study design; number of patients; patient characteristics; marker investigated; cut-off value used; method for determining cut-off value; and numbers in pCR and non-pCR groups. Lead authors were contacted by e-mail and a request for further unpublished data was made when relevant data were not available in the original articles. The STROBE checklist^15^ was used to assess the quality of eligible studies; scores are included in *Table S1*.

### Statistical analysis

A meta-analysis was undertaken of the studies for each inflammatory marker where three or more studies reported pCR rates in relation to the inflammatory marker. Outcome data were reported as ORs. The 95% confidence intervals were estimated using the Mantel–Haenszel method. An OR of greater than 1.00 favoured low levels of the inflammatory marker in achieving a pCR. Individual study results and pooled estimates are displayed in forest plots. Heterogeneity was investigated by visual inspection of forest plots and calculation of the *I*^2^ statistic, which provides the percentage variability attributed to heterogeneity rather than sampling error. A random-effects model was used where *I*^2^ exceeded 50% on the assumption that there was considerable variation between studies. *P* < 0.050 was considered statistically significant. Statistical analysis was performed using Review Manager (RevMan) version 5.3 (Nordic Cochrane Centre, Copenhagen, Denmark).

## Results

**Fig. 1:**
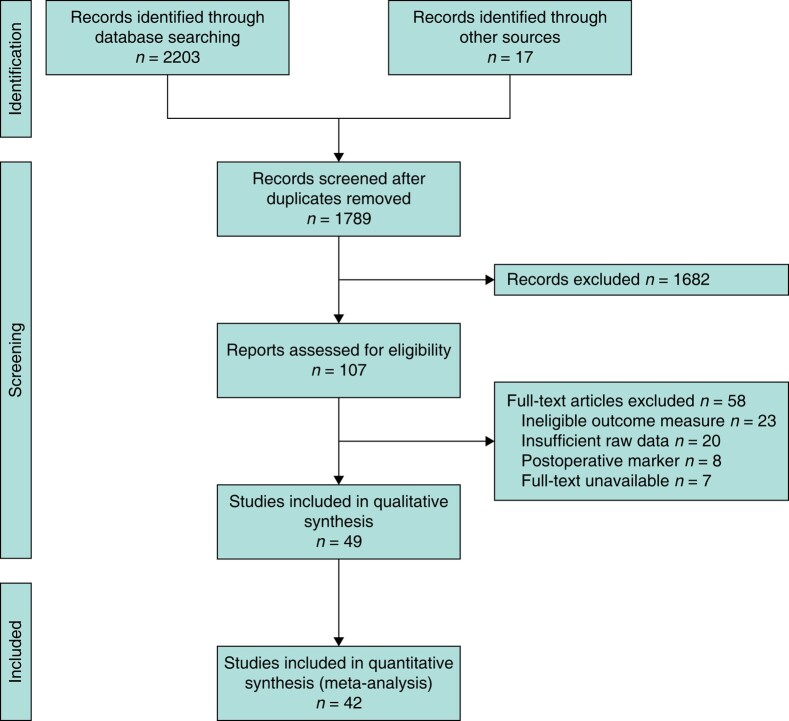
Flow diagram.

The initial search strategy yielded 1789 articles, of which 107 were deemed eligible for review. After application of inclusion and exclusion criteria, 49 studies were selected for inclusion (*Fig. 1*). Of these, 42 studies were suitable for inclusion in the meta-analysis. Individual study details and results are reported in the *Supplementary material*. In the interest of conciseness, only the results of the meta-analysis are described in the main text.

### Predictive role of the pretreatment neutrophil-to-lymphocyte ratio

A total of 34 studies^16–49^ investigated the association between the pretreatment NLR and achieving a pCR after neoadjuvant therapy. Of these studies, 19 included all molecular subtypes of breast cancer and the other 15 included only various specific subtypes. A total of 27 studies used a cut-off value of between 1.63 and 2.74^16–42^ and 7 studies used a cut-off value of greater than or equal to 3^43–49^.

**Fig. 2:**
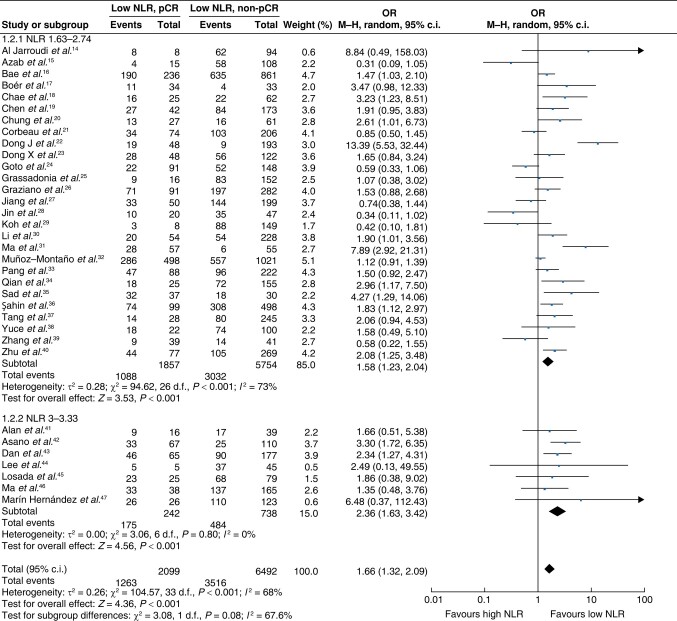
Meta-analysis of the association between the neutrophil-to-lymphocyte ratio and a pCR NLR, neutrophil-to-lymphocyte ratio; M–H, Mantel–Haenszel.

In the meta-analysis of all eligible studies using a pCR as an outcome, 27 studies with 7611 patients were grouped into NLR less than 3, and 7 studies with 980 patients were grouped into NLR greater than or equal to 3 (*Fig. 2*). Both groups demonstrated a significant association between a low NLR and achieving a pCR. This was also the case when they were combined (8591 patients) (pooled OR 1.66 (95% c.i. 1.32 to 2.09); *P* < 0.001), although there was considerable heterogeneity between studies (*I*^2^ = 68%; *P* < 0.001) (*Fig. 2*).

### Predictive role of the pretreatment white cell count and its components

A total of seven studies investigated the WCC or its individual components (*Tables S3–S6*)^19,23,24,32,33,36,50^; one study included only patients with HER2+ breast cancer^19^, whereas the others included all subtypes.

**Fig. 3:**
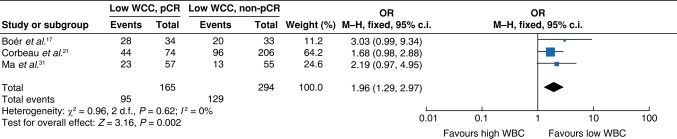
Meta-analysis of the association between the white cell count and a pCR WCC, white cell count; M–H, Mantel–Haenszel.

A total of three studies^19,23,33^ were eligible for meta-analysis of the association between the WCC (using a cut-off value of between 6.13 × 10^9^/l and 8.66 × 10^9^/l) and a pCR. The meta-analysis suggested that patients with a low pretreatment WCC were more likely to achieve a pCR (OR 1.96 (95% c.i. 1.29 to 2.97); *P* = 0.002) (*Fig. 3*).

**Fig. 4:**
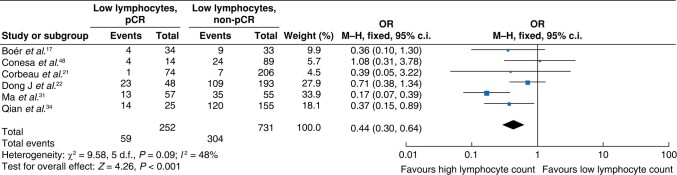
Meta-analysis of the association between the lymphocyte count and a pCR M–H, Mantel–Haenszel.

A total of six studies^19,23,24,33,36,50^ were suitable for the meta-analysis of the lymphocyte count, including 983 patients, of which 252 achieved a pCR. These studies indicated an association between a high lymphocyte count and a pCR (OR 0.44 (95% c.i. 0.30 to 0.64); *P* < 0.0001) (*Fig. 4*).

A total of four studies^19,23,33,36^ investigated the association between the neutrophil count and a pCR. Meta-analysis of these four studies (629 patients with 190 pCR events) favoured a low neutrophil count for achieving a pCR; however, this association was not significant (OR 1.62 (95% c.i. 0.96 to 2.74); *P* = 0.07) (*Fig. S1*).

**Fig. 5:**
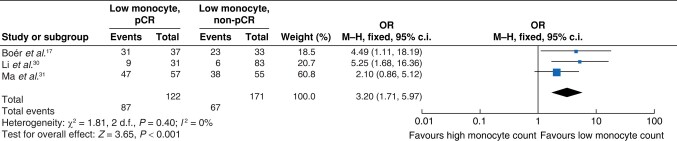
Meta-analysis of the association between the monocyte count and a pCR M–H, Mantel–Haenszel.

Monocytes were another component of the WCC investigated in three studies^19,32,33^ (293 patients with 122 pCR events). Meta-analysis of these studies suggested a significant association between a low pretreatment monocyte count and a pCR (OR 3.20 (95% c.i. 1.71 to 5.97); *P* < 0.001) (*Fig. 5*).

### Predictive role of the pretreatment platelet-to-lymphocyte ratio

**Fig. 6:**
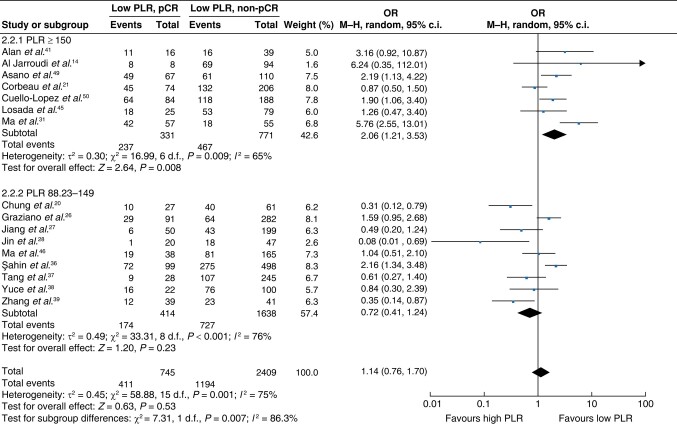
Meta-analysis of the association between the platelet-to-lymphocyte ratio and a pCR PLR, platelet-to-lymphocyte ratio; M–H, Mantel–Haenszel.

A total of 16 studies^16,22,23,28–30,33,38–41,43,47,48,51,52^ evaluated the value of the PLR in predicting a pCR to neoadjuvant therapy. Cut-off values ranged between 88.23 and 225.3. Of the studies, 12 studies included all molecular subtypes^23,28–30,33,38,40,43,47,48,51,52^, 2 studies included only TNBC^22,41^, 1 study included only oestrogen receptor+, HER2− breast cancer^39^, and 1 study included only inflammatory breast cancer^16^. The relationship between the PLR and a pCR was investigated by meta-analysis of the 16 studies, including 3154 patients. There was substantial heterogeneity between studies (*I*^2^ = 75%; *P* < 0.001) and a range of cut-off values was used. No significant association was found between the PLR and a pCR on combined analysis (pooled OR 1.14 (95% c.i. 0.76 to 1.70); *P* = 0.53). In a subgroup analysis using a PLR cut-off value of greater than or equal to 150^16,23,33,43,47,51,52^, a significant association between a low PLR and a pCR (OR 2.06 (95% c.i. 1.21 to 3.53); *P* = 0.008) was shown (*Fig. 6*).

### Predictive role of the pretreatment lymphocyte-to-monocyte ratio

A total of nine studies^24,26,33,37,41,47–49,53^ investigated the predictive role of the LMR in breast cancer patients undergoing neoadjuvant therapy; seven of the studies included all breast cancer subtypes, whereas two of the studies included only TNBC. Cut-off values ranged between 4.62 and 6.39. Meta-analysis of the nine eligible studies, including 1386 patients, showed no significant association between pretreatment LMR values and a pCR (OR 0.67 (95% c.i. 0.36 to 1.24); *P* = 0.20) (*Fig. S2*). Considerable heterogeneity was also found between the studies (*I*^2^ = 79%; *P* < 0.001).

### Predictive role of the pretreatment systemic immune-inflammation index

A total of five studies^29,33,42,43,54^ investigated the role of the SII in predicting the response to neoadjuvant chemotherapy. The SSI = (neutrophil count × platelet count)/lymphocyte count. Cut-off values ranged between 547 and 963. Meta-analysis of the 1555 patients evaluated in these five studies demonstrated no statistically significant association between the SII and a pCR (OR 1.29 (95% c.i. 0.70 to 2.35); *P* = 0.42) and there was substantial heterogeneity between studies (*I*^2^ = 73%; *P* = 0.42) (*Fig. S3*).

### Predictive role of the pretreatment systemic inflammation response index

A total of four studies^24,29,42,54^ investigated the predictive value of the SIRI in the neoadjuvant treatment of breast cancer. These studies included 1260 patients in total, with 235 pCR events. The SIRI = (neutrophil count × monocyte count)/lymphocyte count. Cut-off values ranged between 0.52 and 0.85. Meta-analysis of these five studies showed that a low SIRI tended to favour achieving a pCR, although not to a statistically significant extent (OR 1.41 (95% c.i. 0.65 to 3.04); *P* = 0.38). Considerable heterogeneity was found between studies (*I*^2^ = 84%; *P* < 0.001) (*Fig. S4*).

### Predictive role of pretreatment fibrinogen

A total of four studies^32,39,41,55^ investigated the association between fibrinogen and the response to neoadjuvant therapy. Meta-analysis of these studies (1471 patients) did not demonstrate a significant association between pretreatment fibrinogen levels and pCR rates (OR 1.11 (95% c.i. 0.41 to 3.04); *P* = 0.84). There was significant heterogeneity between studies (*I*^2^ = 83%; *P* < 0.001) and variation between cut-off values (ranging between 1.73 g/L and 3.4 g/L) (*Fig. S5*).

### Predictive role of pretreatment albumin

A total of three studies^32,33,56^ investigated the value of albumin in predicting a pCR in breast cancer. These three studies were included in the meta-analysis, with a total of 772 patients and 162 with a pCR. This failed to show a significant association between albumin and a pCR (OR 0.66 (95% c.i. 0.23 to 1.89); *P* = 0.44). There was considerable heterogeneity between studies (*I*^2^ = 74%; *P* = 0.02) (*Fig. S6*). Notably, cut-off values were similar between studies, ranging between 40 g/L and 52.15 g/L.

### Predictive role of other markers of systemic inflammation

A total of ten studies^23,32,33,38,40,41,43,47,56,57^ evaluated other markers of systemic inflammation in the prediction of response to neoadjuvant therapy in breast cancer, but were unsuitable for meta-analysis due to the limited number of studies for each marker (*Table S13*). There were two studies that investigated pretreatment platelets. Ma *et al*.^33^ found a significant association between a high platelet count and a pCR (*P* = 0.037), whereas Corbeau *et al*.^23^ did not find a statistically significant association between the two (*P* = 0.068). There were two other studies that evaluated pretreatment D-dimer levels, both of which found a statistically significant association between high D-dimer levels and a pCR^32,41^. Şahin *et al*.^38^ included the monocyte-to-lymphocyte ratio (MLR) in their analyses, finding that a low MLR was significantly associated with achieving a pCR on univariable analysis (OR 2.06 (95% c.i. 1.32 to 3.21); *P* = 0.002), but not on multivariable analysis. Şahin *et al*.^38^ also investigated the pan-immune-inflammation-value (PIV), which was calculated by multiplying the neutrophil count by the platelet count and the monocyte count and dividing the result by the lymphocyte count; the study found a significant association between a low PIV and a pCR on both univariable analysis and multivariable analysis (*P* = 0.002). There were two studies that investigated the neutrophil-to-monocyte ratio, with neither finding a significant association with a pCR after neoadjuvant therapy^47,49^. Yuce *et al*.^40^ investigated pretreatment haemoglobin-albumin-lymphocyte-platelet scores and found no significant relationship between them and a pCR (*P* = 0.64). Jiang *et al*.^57^ evaluated the predictive value of a modified systemic inflammation score (mSIS) and found no statistically significant association between the mSIS and a pCR (*P* = 0.379). Zhang *et al*.^41^ evaluated the relationship between the CRP-to-albumin ratio and a pCR and found no association between them. Alan *et al*.^43^ failed to show a significant relationship between pretreatment CRP values and a pCR. Finally, Qu *et al*.^56^ reported a statistically significant association between the albumin-to-alkaline phosphatase ratio and a pCR.

## Discussion

Neoadjuvant systemic therapy for breast cancer has evolved dramatically in recent years. As the indications have expanded, there is increasing demand to find a reliable method of identifying patients most likely to benefit from treatment. This systematic review and meta-analysis evaluated serum inflammatory markers routinely measured before commencing neoadjuvant therapy. The results suggest that the NLR, WCC, lymphocyte count, monocyte count, and PLR may have value in predicting a pCR to neoadjuvant treatment in breast cancer. While there are studies that have investigated individual markers, to the best of the authors’ knowledge, this is the first study to compare the predictive role of a number of serum inflammatory markers in the neoadjuvant treatment of breast cancer.

The correlation between the NLR and a pCR to neoadjuvant therapy has been investigated in recent meta-analyses, including numerous cancer types^10,58,59^. A study investigated the value of the NLR in predicting a pCR after neoadjuvant therapy in multiple solid tumours and found a significant association between the two, although not in the breast cancer subgroup^9^. The results of the present study are supported by a meta-analysis conducted by Cullinane *et al*.^8^, which, although examining fewer studies and patients, found that a low NLR predicted a pCR to neoadjuvant therapy in breast cancer. There is limited evidence to support the role of the NLR in predicting a pCR, but the prognostic role of the NLR in breast cancer has been well investigated, with two recent meta-analyses showing it to be associated with both overall survival (OS) and disease-free survival^6,10^.

While scores derived from components of the WCC have shown predictive value, individual components in isolation have not been well investigated. The present meta-analysis found a significant association between a low pretreatment WCC and a pCR, as well as between a low pretreatment monocyte count and a pCR, albeit with a limited number of studies available. Interestingly, a significant correlation was found between a high pretreatment lymphocyte count and a pCR. A recent meta-analysis investigating the prognostic role of preoperative serum markers in breast cancer found no association between the pretreatment lymphocyte count and OS^6^. This suggests that the pretreatment lymphocyte count may be more useful in predicting the response to neoadjuvant therapy than predicting breast cancer prognosis.

While the PLR has been shown to have prognostic value in breast cancer^60–62^, its role in predicting a pCR to neoadjuvant therapy has been less well studied. A meta-analysis by Long *et al*.^63^ suggested that a lower PLR was associated with an increased likelihood of a pCR, in combined analysis of breast and rectal tumours. This is the first systematic review to investigate the role of the PLR in predicting a pCR, specifically in breast cancer. There was no significant association between the PLR and a pCR on combined analysis; however, a low PLR was associated with a pCR on subgroup analysis of studies using cut-off values greater than or equal to 150 (7 studies).

Novel scores, such as the SII and the SIRI, have been the subject of increasing interest in breast cancer, given their prognostic value in other cancer types^64–67^. The LMR is another interesting marker, with a recent meta-analysis finding an improved prognosis in breast cancer patients with a low LMR^68^. Other novel scores, such as the PIV^38^ and an mSIS^57^, are currently being evaluated; however, both their prognostic value and predictive value are yet to be determined^69^. Dynamic changes in the levels of inflammatory blood markers throughout neoadjuvant treatment have also been suggested to predict a pCR in breast cancer^40,45^, which highlights another area that requires further investigation.

While serum inflammatory markers are unlikely to be of much clinical value in isolation, they could serve as a useful adjunct to clinicopathological and imaging features to identify patients with the highest likelihood of achieving a pCR and thus optimal candidates for neoadjuvant therapy. Furthermore, there has been growing interest in the possibility of omitting surgery in patients with an exceptional response to neoadjuvant therapy. A recent trial by Kuerer *et al*.^70^ reported that this was feasible in patients with a good response to neoadjuvant therapy on imaging and no residual disease on vacuum-assisted core biopsy (VACB) of the tumour bed, with the patients only receiving radiotherapy. Another study showed that a machine-learning algorithm, using patient, imaging, tumour, and VACB variables, could reliably exclude residual disease after neoadjuvant treatment^71^. Further studies are being designed to validate these findings^72^. Given the predictive value of certain inflammatory markers or scores, demonstrated in this meta-analysis, coupled with their convenience, they may serve as useful adjuncts to more accurately predict patients with a high probability of a pCR in whom surgery may be safely omitted.

Serum markers are available through routine, easily accessible blood tests and may therefore be valuable for predicting a pCR, without requiring any additional resources. However, there are many potential limitations, which may prevent their widespread adoption in clinical practice. For example, one such limitation is the wide range of cut-off values used for each inflammatory marker. This may be, at least in part, due to the variability in the methods used to determine cut-off values between studies. Whereas the majority of studies used receiver operating characteristic (ROC) curves to determine threshold values, some used median values, values based on previous literature, laboratory reference ranges, or the third quartile value as their cut-off points. Of note, Asian studies were inclined to use lower cut-off values, which may indicate the effect of ethnicity on inflammation levels^26,29,33^. Furthermore, there are many potential confounders that may limit the predictive value of these markers, such as concurrent illness or systemic inflammatory conditions.

The present systematic review and meta-analysis has several limitations. All of the studies included were retrospective in nature, rendering the data subject to the inherent limitations of incomplete data and potential confounders. Given their potential to influence serum inflammation markers, some studies excluded patients with concurrent illness or systemic inflammatory diseases, whereas others did not. There was also heterogeneity in the molecular subtypes included, with most studies including all breast cancers and others including only specific molecular subtypes. Neoadjuvant treatment regimens also varied between studies as a result of this. While this heterogeneity does limit the ability to derive definitive clinical value from the meta-analysis, the overall value of these circulating inflammatory markers in predicting a pCR remains significant. It is essential that future studies include sufficient detail on tumour molecular subtypes and neoadjuvant treatment regimens to allow the establishment of which patient cohort would derive the greatest clinical value from these markers. Another limitation is that, whereas most studies defined a pCR, some did not. Most studies defined a pCR as the absence of invasive carcinoma both in the breast and the axilla, regardless of the presence of carcinoma *in situ* (ypT0/Tis ypN0); however, one study failed to include the axilla in their definition^73^.

The present systematic review and meta-analysis found the pretreatment NLR, WCC, lymphocyte count, and monocyte count of value in the prediction of a pCR in the neoadjuvant treatment of breast cancer. Further research is required to determine their value in specific breast cancer subtypes and to establish optimal cut-off values, before their adoption in clinical practice.

## Supplementary Material

znae132_Supplementary_Data

## Data Availability

Data used in this study can be made available on request to the corresponding author.
