# Supplementary material for: Predictive value of pretreatment circulating inflammatory response markers in the neoadjuvant treatment of breast cancer: meta-analysis
Source: Br J Surg. 2024 May 27;111(5):znae132. doi: 10.1093/bjs/znae132 (PMC11129713; doi:10.1093/bjs/znae132)
Supplement: znae132_Supplementary_Data [file znae132_supplementary_data.docx]

Table S1 STROBE (Strengthening The Reporting of Observational Studies in Epidemiology) Statement checklist scores for included studies

| Author | Year | STROBE Score (n/22) |
| --- | --- | --- |
| Alan^41^ | 2020 | 18 |
| Al Jarroudi^14^ | 2021 | 21 |
| Asano^49^ | 2016 | 15 |
| Asano^42^ | 2016 | 16 |
| Azab^15^ | 2021 | 19 |
| Bae^16^ | 2020 | 18 |
| Boér^17^ | 2021 | 20 |
| Chae^18^ | 2018 | 19 |
| Chen^19^ | 2016 | 19 |
| Chen^52^ | 2020 | 18 |
| Corbeau^21^ | 2020 | 15 |
| Cuello-López^50^ | 2018 | 18 |
| Chung^20^ | 2022 | 19 |
| Dan^43^ | 2020 | 17 |
| Dong, X.^23^ | 2021 | 18 |
| Dong, J.^22^ | 2021 | 18 |
| Goto^24^ | 2018 | 17 |
| Grassadonia^25^ | 2021 | 18 |
| Graziano^26^ | 2019 | 15 |
| Marín Hernández^47^ | 2018 | 17 |
| Hu^71^ | 2020 | 16 |
| Jiang^27^ | 2020 | 17 |
| Jiang^55^ | 2022 | 18 |
| Jiang^72^ | 2022 | 17 |
| Jin^28^ | 2022 | 19 |
| Koh^29^ | 2014 | 16 |
| Lee^44^ | 2019 | 18 |
| Li^30^ | 2021 | 16 |
| Li^73^ | 2022 | 19 |
| Losada^45^ | 2018 | 17 |
| Lou^74^ | 2022 | 16 |
| Ma^31^ | 2023 | 16 |
| Ma^46^ | 2021 | 18 |
| Meng^51^ | 2022 | 20 |
| Muñoz-Montaño^32^ | 2020 | 18 |
| Pang^33^ | 2021 | 17 |
| Qian^34^ | 2018 | 19 |
| Qu^54^ | 2021 | 18 |
| Sad^35^ | 2020 | 15 |
| Sahin^36^ | 2021 | 18 |
| Tang^37^ | 2022 | 18 |
| Vicente-Conesa^48^ | 2012 | 17 |
| Wang^53^ | 2021 | 19 |
| Yuce^38^ | 2023 | 14 |
| Zhang^39^ | 2019 | 17 |
| Zhu^40^ | 2021 | 19 |
| Zhu^75^ (SII) | 2022 | 16 |
| Zhu^76^ (SIRI) | 2022 | 17 |

Table S2 Studies investigating the predictive role of pre-treatment neutrophil-to-lymphocyte ratio (NLR)

| **Author** | **Year** | **Country** | **Type of study** | **Number of patients** | **Median age** | **Total pCR** | **Cancer stage** | **Cancer subtype** | **ER+** | **PR+** | **HER2+** | **TNBC** | **No pts NLR >threshold** | **Marker** | **Cut-off** | **Method for cut-off** | **pCR defined** |
| --- | --- | --- | --- | --- | --- | --- | --- | --- | --- | --- | --- | --- | --- | --- | --- | --- | --- |
| Koh^29^ | 2014 | South Korea | Retrospective | 157 | 44 | 8 | I - III | Luminal A | 157 | 157 | 0 | 0 | 66 | NLR | 2.25 | ROC | Yes |
| Asano^42^ | 2016 | Japan | Retrospective | 177 | 44 | 67 | II - III | All | - | - | - | 61 | 119 | NLR | 3 | Previous studies | Yes |
| Chen^19^ | 2016 | China | Retrospective | 215 | 46.4 | 42 | II - III | All | 150 | 142 | 77 | 18 | 104 | NLR | 2.1 | ROC | Yes |
| Chae^18^ | 2018 | South Korea | Retrospective | 87 | 45.8 | 25 | I - III | TNBC | 0 | 0 | 0 | 87 | 49 | NLR | 1.7 | ROC | Yes |
| Goto^24^ | 2018 | Japan | Retrospective | 239 | - | 91 | II - III | All | - | - | - | 83 | 165 | NLR | 1.63 | ROC | Yes |
| Marín Hernández^47^ | 2018 | Spain | Retrospective | 150 | 49.8 | 26 | I - III | All | 93 | - | 35 | 38 | 13 | NLR | 3.33 | ROC | Yes |
| Qian^34^ | 2018 | China | Retrospective | 180 | 46 | 25 | II - III | All | 84 | - | 30 | 40 | 90 | NLR | 2.15 | Median | Yes |
| Graziano^26^ | 2019 | Italy | Retrospective | 373 | 50 | 91 | I - III | All | - | - | 238 | 62 | 105 | NLR | 2.42 | ROC | Yes |
| Lee^44^ | 2019 | South Korea | Retrospective | 50 | 51 | 5 | I - III | TNBC | 0 | 0 | 0 | 50 | 8 | NLR | 3.16 | ROC | No |
| Losada^45^ | 2019 | Spain | Retrospective | 113 | 71 | 25 | I - III | All | - | - | 8 | 25 | 13 | NLR | 3.33 | ROC | No |
| Zhang^39^ | 2019 | China | Retrospective | 80 | 49.5 | 39 | I - III | TNBC | 0 | 0 | 0 | 80 | 57 | NLR | 1.71 | ROC | Yes |
| Alan^41^ | 2020 | Turkey | Retrospective | 55 | 48.5 | 16 | II - III | All | 40 | 31 | 16 | 8 | 29 | NLR | 3.3 | Not done | Yes |
| Bae^16^ | 2020 | South Korea | Retrospective | 1097 | 47 | 236 | - | HER2- | 625 | 489 | 0 | 459 | 272 | NLR | 2.74 | ROC | Yes |
| Corbeau^21^ | 2020 | France | Retrospective | 280 | 50.3 | 74 | - | All | - | - | 81 | 72 | 143 | NLR | 2 | ROC | Yes |
| Dan^43^ | 2020 | China | Retrospective | 316 | 50 | 65 | II - III | All | - | - | 41 | 60 | 106 | NLR | 3.1 | Not done | Yes |
| Jiang^27^ | 2020 | China | Retrospective | 249 | 51 | 50 | - | All | 163 | 131 | 88 | 36 | 72 | NLR | 2.13 | ROC | No |
| Muñoz-Montaño^32^ | 2020 | Mexico | Retrospective | 1519 | 49 | 498 | I - III | All | - | - | 377 | 261 | 676 | NLR | 2 | ROC | Yes |
| Sad^35^ | 2020 | Egypt | Retrospective | 67 | 47 | 37 | II - III | TNBC | 0 | 0 | 0 | 67 | 17 | NLR | 1.7 | ROC | Yes |
| Al Jarroudi^14^ | 2021 | Morocco | Retrospective | 102 | 49 | 8 | - | Inflammatory breast cancer | - | - | 18 | - | 32 | NLR | 2.28 | ROC | Yes |
| Azab^15^ | 2021 | USA | Retrospective | 123 | - | 15 | I - III | TNBC | 0 | 0 | 0 | 123 | 61 | NLR | 2 | On basis of previous studies | Yes |
| Boér^17^ | 2021 | Hungary | Retrospective | 82 | 50.3 | 34 | II - III | HER2+ | 32 | 20 | 82 | 0 | 50 | NLR | 1.976 | Not done | Yes |
| Dong J^22^ | 2021 | China | Retrospective | 241 | 48 | 48 | - | All | - | - | 87 | 53 | 213 | NLR | 1.77 | ROC | Yes |
| Dong X^23^ | 2021 | China | Retrospective | 179 | - | 48 | - | TNBC | 179 | 0 | 0 | 179 | 86 | NLR | 1.982 | Not done | Yes |
| Grassadonia^25^ | 2021 | Italy | Retrospective | 168 | 50 | 16 | I - III | Luminal A & Luminal B | - | - | 0 | 0 | 76 | NLR | 2.12 | ROC | yes |
| Li^30^ | 2021 | China | Retrospective | 282 | - | 54 | I - III | All | - | - | 25 | 33 | 208 | NLR | 1.8 | ROC | Yes |
| Ma^46^ | 2021 | China | Retrospective | 203 | 46.6 | 38 | II - III | All | 121 | 114 | 63 | 42 | 33 | NLR | 3 | ROC | Yes |
| Pang^33^ | 2021 | China | Retrospective | 395 | - | 88 | - | TNBC | 0 | 0 | 0 | 395 | 167 | NLR | 1.928 | Median | Yes |
| Şahin^36^ | 2021 | Turkey | Retrospective | 743 | 48 | 99 | - | All | 484 | - | 160 | - | 215 | NLR | 2.34 | ROC | Yes |
| Zhu^40^ | 2021 | China | Retrospective | 346 | 48 | 77 | II - III | All | - | - | 140 | 63 | 197 | NLR | 1.695 | ROC | Yes |
| Chung^20^ | 2022 | Taiwan | Retrospective | 88 | - | 27 | II - III | TNBC | 0 | 0 | 0 | 88 | 59 | NLR | 1.909 | ROC | Yes |
| Jin^28^ | 2022 | China | Retrospective | 67 | 51 | 20 | - | All | - | - | 21 | 12 | 22 | NLR | 2.464 | ROC | Yes |
| Tang^37^ | 2022 | China | Retrospective | 273 | 49.77 | 28 | - | ER+, HER2- | 273 | - | 0 | 0 | 179 | NLR | 2.46 | ROC | Yes |
| Ma^31^ | 2023 | China | Retrospective | 112 | 50.9 | 57 | - | All | 59 | 65 | 90 | 7 | 78 | NLR | 2.02 | ROC | No |
| Yuce^38^ | 2023 | Turkey | Retrospective | 127 | 50.3 | 22 | - | All | - | - | 14 | 10 | 30 | NLR | 2.495 | 3rd quartile | No |

Table S3 Studies investigating the predictive role of pre-treatment white cell count (WCC)

| **Author** | **Year** | **Country** | **Type of study** | **Number of patients** | **Median age** | **Total pCR** | **Cancer stage** | **Cancer subtype** | **ER+** | **PR+** | **HER2+** | **TNBC** | **No pts WCC >threshold** | **Marker** | **Cut-off** | **Method for cut-off** | **pCR defined** |
| --- | --- | --- | --- | --- | --- | --- | --- | --- | --- | --- | --- | --- | --- | --- | --- | --- | --- |
| Corbeau^21^ | 2020 | France | Retrospective | 280 | 50.3 | 74 | - | All | - | - | 81 | 72 | 140 | WCC | 6.75 | ROC | Yes |
| Boér^17^ | 2021 | Hungary | Retrospective | 82 | 50.3 | 34 | II - III | HER2+ | 32 | 20 | 82 | 0 | 24 | WCC | 8.66 | Not done | Yes |
| Ma^31^ | 2023 | China | Retrospective | 112 | 50.9 | 57 | - | All | 59 | 65 | 90 | 7 | 77 | WCC | 6.13 | ROC | No |

Table S4 Studies investigating the predictive role of pre-treatment lymphocytes

| **Author** | **Year** | **Country** | **Type of study** | **Number of patients** | **Median age** | **Total pCR** | **Cancer stage** | **Cancer subtype** | **ER+** | **PR+** | **HER2+** | **TNBC** | **No pts lymphocyte >threshold** | **Marker** | **Cut-off** | **Method for cut-off** | **pCR defined** |
| --- | --- | --- | --- | --- | --- | --- | --- | --- | --- | --- | --- | --- | --- | --- | --- | --- | --- |
| Conesa^48^ | 2012 | Spain | Retrospective | 103 | 50 | 14 | II - III | All | 85 | 53 | 19 | 22 | 75 | Lymphocytes | 1.5 | Not done | Yes |
| Qian^34^ | 2018 | China | Retrospective | 180 | 46 | 25 | II - III | All | 84 | - | 30 | 40 | 46 | Lymphocytes | 2.06 | ROC | Yes |
| Corbeau^21^ | 2020 | France | Retrospective | 280 | 50.3 | 74 | - | All | - | - | 81 | 72 | 272 | Lymphocytes | 1 | ROC | Yes |
| Boér^17^ | 2021 | Hungary | Retrospective | 82 | 50.3 | 34 | II - III | HER2+ | 32 | 20 | 82 | 0 | 52 | Lymphocytes | 1.62 | Not done | Yes |
| Dong J^22^ | 2021 | China | Retrospective | 241 | 48 | 48 | - | All | - | - | 87 | 53 | 109 | Lymphocytes | 1.57 | ROC | Yes |
| Ma^31^ | 2023 | China | Retrospective | 112 | 50.9 | 57 | - | All | 59 | 65 | 90 | 7 | 64 | Lymphocytes | 1.64 | ROC | No |

Table S5 Studies investigating the predictive role of pre-treatment neutrophils

| **Author** | **Year** | **Country** | **Type of study** | **Number of patients** | **Median age** | **Total pCR** | **Cancer stage** | **Cancer subtype** | **ER+** | **PR+** | **HER2+** | **TNBC** | **No pts neutrophils >threshold** | **Marker** | **Cut-off** | **Method for cut-off** | **pCR defined** |
| --- | --- | --- | --- | --- | --- | --- | --- | --- | --- | --- | --- | --- | --- | --- | --- | --- | --- |
| Qian^34^ | 2018 | China | Retrospective | 180 | 46 | 25 | II - III | All | 84 | - | 30 | 40 | 146 | Neutrophils | 2.65 | ROC | Yes |
| Corbeau^21^ | 2020 | France | Retrospective | 280 | 50.3 | 74 | - | All | - | - | 81 | 72 | 279 | Neutrophils | 1.5 | ROC | Yes |
| Boér^17^ | 2021 | Hungary | Retrospective | 82 | 50.3 | 34 | II - III | HER2+ | 32 | 20 | 82 | 0 | 30 | Neutrophils | 5.4 | Not done | Yes |
| Ma^31^ | 2023 | China | Retrospective | 112 | 50.9 | 57 | - | All | 59 | 65 | 90 | 7 | 54 | Neutrophils | 4.7 | ROC | No |

Table S6 Studies investigating the predictive role of pre-treatment monocytes

| **Author** | **Year** | **Country** | **Type of study** | **Number of patients** | **Median age** | **Total pCR** | **Cancer stage** | **Cancer subtype** | **ER+** | **PR+** | **HER2+** | **TNBC** | **No pts monocytes >threshold** | **Marker** | **Cut-off** | **Method for cut-off** | **pCR defined** |
| --- | --- | --- | --- | --- | --- | --- | --- | --- | --- | --- | --- | --- | --- | --- | --- | --- | --- |
| Boér^17^ | 2021 | Hungary | Retrospective | 82 | 50.3 | 34 | II - III | HER2+ | 32 | 20 | 82 | 0 | 12 | Monocyte | 0.63 | Not done | Yes |
| Li^73^ | 2022 | China | Retrospective | 114 | 47.9 | 31 | II - III | All | 56 | 29 | 87 | 20 | 99 | Monocyte | 0.185 | ROC | Yes |
| Ma^31^ | 2023 | China | Retrospective | 112 | 50.9 | 57 | - | All | 59 | 65 | 90 | 7 | 27 | Monocyte | 0.44 | ROC | No |

Table S7 Studies investigating the predictive role of pre-treatment platelet-to-lymphocyte ratio (PLR)

| **Author** | **Year** | **Country** | **Type of study** | **Number of patients** | **Median age** | **Total pCR** | **Cancer stage** | **Cancer subtype** | **ER+** | **PR+** | **HER2+** | **TNBC** | **No pts PLR >threshold** | **Marker** | **Cut-off** | **Method for cut-off** | **pCR defined** |
| --- | --- | --- | --- | --- | --- | --- | --- | --- | --- | --- | --- | --- | --- | --- | --- | --- | --- |
| Asano^49^ | 2016 | Japan | Retrospective | 177 | - | 67 | II - III | All | - | - | 36 | 61 | 67 | PLR | 150 | On basis of previous studies | Yes |
| Cuello-Lopez^50^ | 2018 | Colombia | Retrospective | 272 | 51 | 84 | II - III | All | 166 | 131 | 99 | 64 | 90 | PLR | 150 | On basis of previous studies | Yes |
| Graziano^26^ | 2019 | Italy | Retrospective | 373 | 50 | 91 | I - III | All | - | - | 238 | 62 | 280 | PLR | 104.47 | ROC | Yes |
| Zhang^39^ | 2019 | China | Retrospective | 80 | 49.5 | 39 | I - III | TNBC | 0 | 0 | 0 | 80 | 45 | PLR | 129.7 | ROC | Yes |
| Losada^45^ | 2019 | Spain | Retrospective | 113 | 71 | 25 | I - III | All | - | - | 8 | 25 | 33 | PLR | 150 | ROC | No |
| Jiang^27^ | 2020 | China | Retrospective | 249 | 51 | 50 | - | All | 163 | 131 | 88 | 36 | 200 | PLR | 88.23 | ROC | No |
| Corbeau^21^ | 2020 | France | Retrospective | 280 | 50.3 | 74 | - | All | - | - | 81 | 72 | 103 | PLR | 150 | ROC | Yes |
| Alan^41^ | 2020 | Turkey | Retrospective | 55 | 48.5 | 16 | II - III | All | 40 | 31 | 16 | 8 | 28 | PLR | 225.3 | Not done | Yes |
| Şahin^36^ | 2021 | Turkey | Retrospective | 743 | 48 | 99 | - | All | 484 | - | 160 | - | 250 | PLR | 131.8 | ROC | Yes |
| Ma^46^ | 2021 | China | Retrospective | 203 | 46.6 | 38 | II - III | All | 121 | 114 | 63 | 42 | 103 | PLR | 135 | ROC | Yes |
| Al Jarroudi^14^ | 2021 | Morocco | Retrospective | 102 | 49 | 8 | - | Inflammatory breast cancer | - | - | 18 | - | 25 | PLR | 178 | ROC | Yes |
| Jin^28^ | 2022 | China | Retrospective | 67 | 51 | 20 | - | All | - | - | 21 | 12 | 48 | PLR | 106.3 | ROC | Yes |
| Tang^37^ | 2022 | China | Retrospective | 273 | 49.77 | 28 | - | ER+, HER2- | 273 | - | 0 | 0 | 157 | PLR | 117.88 | ROC | Yes |
| Chung^20^ | 2022 | Taiwan | Retrospective | 88 | - | 27 | II - III | TNBC | 0 | 0 | 0 | 88 | 38 | PLR | 148.14 | ROC | Yes |
| Yuce^38^ | 2023 | Turkey | Retrospective | 127 | 50.3 | 22 | - | All | - | - | 14 | 10 | 30 | PLR | 144.25 | 3rd quartile | No |
| Ma^31^ | 2023 | China | Retrospective | 112 | 50.9 | 57 | - | All | 59 | 65 | 90 | 7 | 52 | PLR | 161.5 | ROC | No |

Table S8 Studies investigating the predictive role of pre-treatment lymphocyte-to-monocyte ratio (LMR)

| **Author** | **Year** | **Country** | **Type of study** | **Number of patients** | **Median age** | **Total pCR** | **Cancer stage** | **Cancer subtype** | **ER+** | **PR+** | **HER2+** | **TNBC** | **No pts LMR >threshold** | **Marker** | **Cut-off** | **Method for cut-off** | **pCR defined** |
| --- | --- | --- | --- | --- | --- | --- | --- | --- | --- | --- | --- | --- | --- | --- | --- | --- | --- |
| Goto^24^ | 2018 | Japan | Retrospective | 239 | - | 91 | II - III | All | - | - | - | 83 | 119 | LMR | 6 | ROC | Yes |
| Marín Hernández^47^ | 2018 | Spain | Retrospective | 150 | 49.8 | 26 | I - III | All | 93 | - | 35 | 38 | 28 | LMR | 5.46 | ROC | Yes |
| Losada^45^ | 2019 | Spain | Retrospective | 113 | 71 | 25 | I - III | All | - | - | 8 | 25 | 32 | LMR | 5.46 | ROC | No |
| Zhang^39^ | 2019 | China | Retrospective | 80 | 49.5 | 39 | I - III | TNBC | 0 | 0 | 0 | 80 | 43 | LMR | 5.37 | ROC | Yes |
| Sad^35^ | 2020 | Egypt | Retrospective | 67 | 47 | 37 | II - III | TNBC | 0 | 0 | 0 | 67 | 55 | LMR | 5.3 | ROC | Yes |
| Dong J^22^ | 2021 | China | Retrospective | 241 | 48 | 48 | - | All | - | - | 87 | 53 | 40 | LMR | 5.38 | ROC | Yes |
| Ma^46^ | 2021 | China | Retrospective | 203 | 46.6 | 38 | II - III | All | 121 | 114 | 63 | 42 | 134 | LMR | 6.2 | ROC | Yes |
| Meng^51^ | 2022 | China | Retrospective | 192 | 49 | 30 | - | All | - | - | 62 | - | 96 | LMR | 4.62 | Median | Yes |
| Ma^31^ | 2023 | China | Retrospective | 112 | 50.9 | 57 | - | All | 59 | 65 | 90 | 7 | 38 | LMR | 6.39 | ROC | No |

Table S9 Studies investigating the predictive role of pre-treatment systemic immune-inflammation index (SII)

| **Author** | **Year** | **Country** | **Type of study** | **Number of patients** | **Median age** | **Total pCR** | **Cancer stage** | **Cancer subtype** | **ER+** | **PR+** | **HER2+** | **TNBC** | **No pts SII >threshold** | **Marker** | **Cut-off** | **Method for cut-off** | **pCR defined** |
| --- | --- | --- | --- | --- | --- | --- | --- | --- | --- | --- | --- | --- | --- | --- | --- | --- | --- |
| Alan^41^ | 2020 | Turkey | Retrospective | 55 | 48.5 | 16 | II - III | All | 40 | 31 | 16 | 8 | 27 | SII | 963 | Not done | Yes |
| Chen^52^ | 2020 | China | Retrospective | 262 | 48 | 54 | II - III | All | 154 | 133 | 94 | 67 | 106 | SII | 602 | ROC | No |
| Jiang^27^ | 2020 | China | Retrospective | 249 | 51 | 50 | - | All | 163 | 131 | 88 | 36 | 66 | SII | 547 | ROC | No |
| Zhu^75^ | 2022 | China | Retrospective | 477 | 47 | 72 | I - III | All | 286 | 288 | 164 | 113 | 260 | SII | 560 | ROC | No |
| Ma^31^ | 2023 | China | Retrospective | 112 | 50.9 | 57 | - | All | 59 | 65 | 90 | 7 | 67 | SII | 598.5 | ROC | No |

Table S10 Studies investigating the predictive role of pre-treatment systemic inflammation response index (SIRI)

| **Author** | **Year** | **Country** | **Type of study** | **Number of patients** | **Median age** | **Total pCR** | **Cancer stage** | **Cancer subtype** | **ER+** | **PR+** | **HER2+** | **TNBC** | **No pts SIRI >threshold** | **Marker** | **Cut-off** | **Method for cut-off** | **pCR defined** |
| --- | --- | --- | --- | --- | --- | --- | --- | --- | --- | --- | --- | --- | --- | --- | --- | --- | --- |
| Chen^77^ | 2020 | China | Retrospective | 262 | 48 | 54 | II - III | All | 154 | 133 | 94 | 67 | 107 | SIRI | 0.85 | ROC | No |
| Dong J^22^ | 2021 | China | Retrospective | 241 | 48 | 48 | - | All | - | - | 87 | 53 | 178 | SIRI | 0.72 | ROC | Yes |
| Jiang^72^ | 2022 | China | Retrospective | 280 | 49 | 61 | II - III | All | 155 | 126 | 94 | 59 | 213 | SIRI | 0.52 | ROC | No |
| Zhu^76^ | 2022 | China | Retrospective | 477 | 47 | 72 | I - III | All | 286 | 288 | 164 | 113 | 210 | SIRI | 0.8 | ROC | No |

Table S11 Studies investigating the predictive role of pre-treatment fibrinogen

| **Author** | **Year** | **Country** | **Type of study** | **Number of patients** | **Median age** | **Total pCR** | **Cancer stage** | **Cancer subtype** | **ER+** | **PR+** | **HER2+** | **TNBC** | **No pts fibrinogen >threshold** | **Marker** | **Cut-off** | **Method for cut-off** | **pCR defined** |
| --- | --- | --- | --- | --- | --- | --- | --- | --- | --- | --- | --- | --- | --- | --- | --- | --- | --- |
| Zhang^39^ | 2019 | China | Retrospective | 80 | 49.5 | 39 | I - III | TNBC | 0 | 0 | 0 | 80 | 50 | Fibrinogen | 2.92 | ROC | Yes |
| Wang^53^ | 2021 | China | Retrospective | 1004 | 49 | 198 | I - III | All | 600 | 441 | 427 | 155 | 260 | Fibrinogen | 3.435 | ROC | Yes |
| Li^73^ | 2022 | China | Retrospective | 114 | 47.9 | 31 | II - III | All | 56 | 29 | 87 | 20 | 50 | Fibrinogen | 3.085 | ROC | Yes |
| Tang^37^ | 2022 | China | Retrospective | 273 | 49.77 | 28 | - | ER+, HER2- | 273 | - | 0 | 0 | 234 | Fibrinogen | 1.73 | ROC | Yes |

Table S12 Studies investigating the predictive role of pre-treatment albumin

| **Author** | **Year** | **Country** | **Type of study** | **Number of patients** | **Median age** | **Total pCR** | **Cancer stage** | **Cancer subtype** | **ER+** | **PR+** | **HER2+** | **TNBC** | **No pts albumin >threshold** | **Marker** | **Cut-off** | **Method for cut-off** | **pCR defined** |
| --- | --- | --- | --- | --- | --- | --- | --- | --- | --- | --- | --- | --- | --- | --- | --- | --- | --- |
| Qu^54^ | 2021 | China | Retrospective | 546 | 49 | 74 | - | All | 340 | 266 | 237 | 79 | 334 | Albumin | 40 | Normal reference value | Yes |
| Li^73^ | 2022 | China | Retrospective | 114 | 47.9 | 31 | II - III | All | 56 | 29 | 87 | 20 | 31 | Albumin | 47.45 | ROC | Yes |
| Ma^31^ | 2023 | China | Retrospective | 112 | 50.9 | 57 | - | All | 59 | 65 | 90 | 7 | 84 | Albumin | 52.15 | ROC | No |

Table S13 Studies investigating the predictive role of other pre-treatment inflammatory markers

| **Author** | **Year** | **Country** | **Type of study** | **Number of patients** | **Median age** | **Total pCR** | **Cancer stage** | **Cancer subtype** | **ER+** | **PR+** | **HER2+** | **TNBC** | **No pts monocytes >threshold** | **Marker** | **Cut-off** | **Method for cut-off** | **pCR defined** |
| --- | --- | --- | --- | --- | --- | --- | --- | --- | --- | --- | --- | --- | --- | --- | --- | --- | --- |
| Corbeau^21^ | 2020 | France | Retrospective | 280 | 50.3 | 74 | - | All | - | - | 81 | 72 | 139 | Platelets | 264 G/L | ROC | Yes |
| Ma^31^ | 2023 | China | Retrospective | 112 | 50.9 | 57 | - | All | 59 | 65 | 90 | 7 | 68 | Platelets | 247.5 | ROC | No |
| Zhang^39^ | 2019 | China | Retrospective | 80 | 49.5 | 39 | I - III | TNBC | 0 | 0 | 0 | 80 | 67 | D-dimer | 67.5 | ROC | Yes |
| Li^73^ | 2022 | China | Retrospective | 114 | 47.9 | 31 | II - III | All | 56 | 29 | 87 | 20 | 31 | D-dimer | 0.55 mg/L | ROC | Yes |
| Şahin^36^ | 2021 | Turkey | Retrospective | 743 | 48 | 99 | - | All | 484 | - | 160 | - | 305 | MLR | 0.22 | ROC | Yes |
| Yuce^38^ | 2023 | Turkey | Retrospective | 127 | 50.3 | 22 | - | All | - | - | 14 | 10 | 30 | HALP | 60.974 | 3rd quartile | No |
| Şahin^36^ | 2021 | Turkey | Retrospective | 743 | 48 | 99 | - | All | 484 | - | 160 | - | 246 | PIV | 306.4 | ROC | Yes |
| Jiang^55^ | 2022 | China | Retrospective | 305 | 49 | 56 | I - III | All | 169 | 136 | 107 | 60 | 65 | mSIS | 0 = Low risk, 1/2 = High risk |  | Yes |
| Losada^45^ | 2019 | Spain | Retrospective | 113 | 71 | 25 | I - III | All | - | - | 8 | 25 | 43 | NMR | 9.65 | ROC | No |
| Marín Hernández^47^ | 2018 | Spain | Retrospective | 150 | 49.8 | 26 | I - III | All | 93 | - | 35 | 38 | 34 | NMR | 9.65 | ROC | Yes |
| Zhang^39^ | 2019 | China | Retrospective | 80 | 49.5 | 39 | I - III | TNBC | 0 | 0 | 0 | 80 | 35 | CAR | 0.047 | ROC | Yes |
| Alan^41^ | 2020 | Turkey | Retrospective | 55 | 48.5 | 16 | II - III | All | 40 | 31 | 16 | 8 | 17 | CRP | 5 | Not done | Yes |
| Qu^54^ | 2021 | China | Retrospective | 546 | 49 | 74 |  | All | 340 | 266 | 237 | 79 | 313 | AAPR | 0.583 | ROC | Yes |

Figure S1 Meta-analysis of association between neutrophils and pathological complete response

Figure S2 Meta-analysis of association between lymphocyte-to-monocyte ratio and pathological complete response

Figure S3 Meta-analysis of association between systematic immune-inflammation index and pathological complete response

Figure S4 Meta-analysis of association between systemic inflammation response index and pathological complete response

Figure S5 Meta-analysis of association between fibrinogen and pathological complete response

Figure S6 Meta-analysis of association between albumin and pathological complete response

**References**

1. Pathological complete response in neoadjuvant treatment of high-risk early-stage breast cancer: use as an endpoint to support accelerated approval. United States Food and Drug Administration, Oncology Center of Excellence 2020.

2. Cortazar P, Zhang L, Untch M, et al. Pathological complete response and long-term clinical benefit in breast cancer: the CTNeoBC pooled analysis. Lancet 2014;384:164-72.

3. Broglio KR, Quintana M, Foster M, et al. Association of Pathologic Complete Response to Neoadjuvant Therapy in HER2-Positive Breast Cancer With Long-Term Outcomes: A Meta-Analysis. JAMA Oncol 2016;2:751-60.

4. McAllister SS, Weinberg RA. The tumour-induced systemic environment as a critical regulator of cancer progression and metastasis. Nat Cell Biol 2014;16:717-27.

5. Zhao H, Wu L, Yan G, et al. Inflammation and tumor progression: signaling pathways and targeted intervention. Signal Transduction and Targeted Therapy 2021;6:263.

6. Savioli F, Morrow ES, Dolan RD, et al. Prognostic role of preoperative circulating systemic inflammatory response markers in primary breast cancer: meta-analysis. Br J Surg 2022;109:1206-15.

7. Luo G, Guo M, Liu Z, et al. Blood neutrophil-lymphocyte ratio predicts survival in patients with advanced pancreatic cancer treated with chemotherapy. Ann Surg Oncol 2015;22:670-6.

8. Cullinane C, Creavin B, O'Leary DP, et al. Can the Neutrophil to Lymphocyte Ratio Predict Complete Pathologic Response to Neoadjuvant Breast Cancer Treatment? A Systematic Review and Meta-analysis. Clin Breast Cancer 2020;20:e675-e81.

9. Li X, Dai D, Chen B, Tang H, Xie X, Wei W. The value of neutrophil-to-lymphocyte ratio for response and prognostic effect of neoadjuvant chemotherapy in solid tumors: A systematic review and meta-analysis. J Cancer 2018;9:861-71.

10. Ethier JL, Desautels D, Templeton A, Shah PS, Amir E. Prognostic role of neutrophil-to-lymphocyte ratio in breast cancer: a systematic review and meta-analysis. Breast Cancer Res 2017;19:2.

11. Kaimin H, Lixia L, Juan Y, Suzhan Z. Prognostic role of the neutrophil–lymphocyte ratio in renal cell carcinoma: a meta-analysis. BMJ Open 2015;5:e006404.

12. Templeton AJ, McNamara MG, Šeruga B, et al. Prognostic role of neutrophil-to-lymphocyte ratio in solid tumors: a systematic review and meta-analysis. J Natl Cancer Inst 2014;106:dju124.

13. von Elm E, Altman DG, Egger M, Pocock SJ, Gøtzsche PC, Vandenbroucke JP. The Strengthening the Reporting of Observational Studies in Epidemiology (STROBE) statement: guidelines for reporting observational studies. Lancet 2007;370:1453-7.

14. Al Jarroudi O, El Bairi K, Abda N, et al. Neutrophil-to-lymphocyte and platelet-to-lymphocyte ratios as predictors of outcomes in inflammatory breast cancer. Biomarkers in Medicine 2021;15:1289-98.

15. Azab B, Amundson JR, Cioci A, et al. The Usefulness of the Pretreatment Neutrophil/Lymphocyte Ratio as a Predictor of the 5-Year Survival in Stage 1-3 Triple Negative Breast Cancer Patients. Breast Care 2021;16:43-9.

16. Bae SJ, Cha YJ, Yoon C, et al. Prognostic value of neutrophil-to-lymphocyte ratio in human epidermal growth factor receptor 2-negative breast cancer patients who received neoadjuvant chemotherapy. Scientific reports 2020;10:13078.

17. Boér K, Kahán Z, Landherr L, et al. Pathologic Complete Response Rates After Neoadjuvant Pertuzumab and Trastuzumab with Chemotherapy in Early Stage HER2-Positive Breast Cancer - Increasing Rates of Breast Conserving Surgery: A Real-World Experience. Pathology and Oncology Research 2021;27.

18. Chae S, Kang KM, Kim HJ, et al. Neutrophil–lymphocyte ratio predicts response to chemotherapy in triple-negative breast cancer. Current Oncology 2018;25:e113-e9.

19. Chen Y, Chen K, Xiao X, et al. Pretreatment neutrophil-to-lymphocyte ratio is correlated with response to neoadjuvant chemotherapy as an independent prognostic indicator in breast cancer patients: a retrospective study. BMC Cancer 2016;16:320.

20. Chung WS, Chen SC, Ko TM, et al. An Integrative Clinical Model for the Prediction of Pathological Complete Response in Patients with Operable Stage II and Stage III Triple-Negative Breast Cancer Receiving Neoadjuvant Chemotherapy. Cancers 2022;14.

21. Corbeau I, Thezenas S, Maran-Gonzalez A, Colombo PE, Jacot W, Guiu S. Inflammatory blood markers as prognostic and predictive factors in early breast cancer patients receiving neoadjuvant chemotherapy. Cancers 2020;12:1-16.

22. Dong J, Sun Q, Pan Y, Lu N, Han X, Zhou Q. Pretreatment systemic inflammation response index is predictive of pathological complete response in patients with breast cancer receiving neoadjuvant chemotherapy. BMC Cancer 2021;21.

23. Dong X, Liu C, Yuan J, et al. Prognostic roles of neutrophil-to-lymphocyte ratio and stromal tumor-infiltrating lymphocytes and their relationship in locally advanced triple-negative breast cancer treated with neoadjuvant chemotherapy. Breast Care 2021;16:328-34.

24. Goto W, Kashiwagi S, Asano Y, et al. Predictive value of lymphocyte-to-monocyte ratio in the preoperative setting for progression of patients with breast cancer. BMC Cancer 2018;18:1137.

25. Grassadonia A, Graziano V, Iezzi L, et al. Prognostic relevance of neutrophil to lymphocyte ratio (Nlr) in luminal breast cancer: A retrospective analysis in the neoadjuvant setting. Cells 2021;10.

26. Graziano V, Grassadonia A, Iezzi L, et al. Combination of peripheral neutrophil-to-lymphocyte ratio and platelet-to-lymphocyte ratio is predictive of pathological complete response after neoadjuvant chemotherapy in breast cancer patients. Breast 2019;44:33-8.

27. Jiang C, Lu Y, Zhang S, Huang Y. Systemic Immune-Inflammation Index Is Superior to Neutrophil to Lymphocyte Ratio in Prognostic Assessment of Breast Cancer Patients Undergoing Neoadjuvant Chemotherapy. BioMed Research International 2020;2020.

28. Jin X, Wang K, Shao X, Huang J. Prognostic implications of the peripheral platelet-to-lymphocyte ratio and neutrophil-to-lymphocyte ratio in predicting pathologic complete response after neoadjuvant chemotherapy in breast cancer patients. Gland Surgery 2022;11:1057-66.

29. Koh YW, Lee HJ, Ahn JH, Lee JW, Gong G. Prognostic significance of the ratio of absolute neutrophil to lymphocyte counts for breast cancer patients with ER/PR-positivity and HER2-negativity in neoadjuvant setting. Tumour Biol 2014;35:9823-30.

30. Li X, Tan Q, Li H, Yang X. Predictive value of pretreatment peripheral neutrophil-to-lymphocyte ratio for response to neoadjuvant chemotherapy and breast cancer prognosis. Cancer Management and Research 2021;13:5889-98.

31. Ma R, Wei W, Ye H, Dang C, Li K, Yuan D. A nomogram based on platelet-to-lymphocyte ratio for predicting pathological complete response of breast cancer after neoadjuvant chemotherapy. BMC Cancer 2023;23.

32. Muñoz-Montaño W, Cabrera-Galeana P, Alvarado-Miranda A, et al. Prognostic Value of the Pretreatment Neutrophil-to-Lymphocyte Ratio in Different Phenotypes of Locally Advanced Breast Cancer During Neoadjuvant Systemic Treatment. Clinical Breast Cancer 2020;20:307-16.e1.

33. Pang J, Zhou H, Dong X, Wang S, Xiao Z. Relationship Between the Neutrophil to Lymphocyte Ratio, Stromal Tumor-infiltrating Lymphocytes, and the Prognosis and Response to Neoadjuvant Chemotherapy in Triple-negative Breast Cancer. Clinical Breast Cancer 2021;21:e681-e7.

34. Y Q, J T, X L, et al. Peripheral inflammation/immune indicators of chemosensitivity and prognosis in breast cancer patients treated with neoadjuvant chemotherapy. OncoTargets and therapy 2018;11:1423-32.

35. Sad LM, Elsaka A, Zamzam Y, Almorsy WA. Local and systemic inflammatory markers as prognostic and predictive markers in locally advanced triple negative breast cancer. Onkologia i Radioterapia 2020;14:27-34.

36. AB Ş, E C, B O, et al. Low pan-immune-inflammation-value predicts better chemotherapy response and survival in breast cancer patients treated with neoadjuvant chemotherapy. Scientific reports 2021;11:14662.

37. Tang L, Shu X, Tu G. Exploring the influencing factors of the pathologic complete response in estrogen receptor-positive, HER2-negative breast cancer after neoadjuvant chemotherapy: a retrospective study. World Journal of Surgical Oncology 2022;20.

38. Yuce E, Karakullukcu S, Bulbul H, Alandag C, Saygin I, Kavgaci H. The effect of the change in hemoglobin-albumin-lymphocyte-platelet scores occurring with neoadjuvant chemotherapy on clinical and pathological responses in breast cancer. Bratislavske lekarske listy 2023;124:59-63.

39. Zhang F, Huang M, Zhou H, et al. A Nomogram to Predict the Pathologic Complete Response of Neoadjuvant Chemotherapy in Triple-Negative Breast Cancer Based on Simple Laboratory Indicators. Annals of Surgical Oncology 2019;26:3912-9.

40. Zhu J, Jiao D, Zhao Y, et al. Development of a predictive model utilizing the neutrophil to lymphocyte ratio to predict neoadjuvant chemotherapy efficacy in early breast cancer patients. Scientific reports 2021;11:1350.

41. Alan O, Akin Telli T, Aktas B, et al. Is insulin resistance a predictor for complete response in breast cancer patients who underwent neoadjuvant treatment? World Journal of Surgical Oncology 2020;18.

42. Asano Y, Kashiwagi S, Onoda N, et al. Predictive Value of Neutrophil/Lymphocyte Ratio for Efficacy of Preoperative Chemotherapy in Triple-Negative Breast Cancer. Ann Surg Oncol 2016;23:1104-10.

43. Dan J, Tan J, Huang J, et al. The dynamic change of neutrophil to lymphocyte ratio is predictive of pathological complete response after neoadjuvant chemotherapy in breast cancer patients. Breast Cancer 2020;27:982-8.

44. Lee J, Kim DM, Lee A. Prognostic role and clinical association of tumor-infiltrating lymphocyte, programmed death ligand-1 expression with neutrophil-lymphocyte ratio in locally advanced triple-negative breast cancer. Cancer Research and Treatment 2019;51:649-63.

45. Losada B, Guerra JA, Malón D, Jara C, Rodriguez L, Del Barco S. Pretreatment neutrophil/lymphocyte, platelet/lymphocyte, lymphocyte/monocyte, and neutrophil/monocyte ratios and outcome in elderly breast cancer patients. Clin Transl Oncol 2019;21:855-63.

46. Ma Y, Zhang J, Chen X. Lymphocyte-to-monocyte ratio is associated with the poor prognosis of breast cancer patients receiving neoadjuvant chemotherapy. Cancer Management and Research 2021;13:1571-80.

47. Marín Hernández C, Piñero Madrona A, Gil Vázquez PJ, et al. Usefulness of lymphocyte-to-monocyte, neutrophil-to-monocyte and neutrophil-to-lymphocyte ratios as prognostic markers in breast cancer patients treated with neoadjuvant chemotherapy. Clinical and Translational Oncology 2018;20:476-83.

48. Vicente Conesa MA, Garcia-Martinez E, Gonzalez Billalabeitia E, et al. Predictive value of peripheral blood lymphocyte count in breast cancer patients treated with primary chemotherapy. Breast 2012;21:468-74.

49. Asano Y, Kashiwagi S, Onoda N, et al. Platelet–lymphocyte ratio as a useful predictor of the therapeutic effect of neoadjuvant chemotherapy in breast cancer. PLoS ONE 2016;11.

50. Cuello-López J, Fidalgo-Zapata A, López-Agudelo L, Vásquez-Trespalacios E. Platelet-To-lymphocyte ratio as a predictive factor of complete pathologic response to neoadjuvant chemotherapy in breast cancer. PLoS ONE 2018;13.

51. Meng X, Wang X, Jiang C, Zhang S, Cheng S. Correlation analysis of lymphocyte-monocyte ratio with pathological complete response and clinical prognosis of neoadjuvant chemotherapy in patients with breast cancer. Translational Oncology 2022;18.

52. Chen L, Kong X, Wang Z, Wang X, Fang Y, Wang J. Pre-treatment systemic immune-inflammation index is a useful prognostic indicator in patients with breast cancer undergoing neoadjuvant chemotherapy. Journal of Cellular and Molecular Medicine 2020;24:2993-3021.

53. Y W, Y W, R C, et al. Plasma fibrinogen acts as a predictive factor for pathological complete response to neoadjuvant chemotherapy in breast cancer: a retrospective study of 1004 Chinese breast cancer patients. BMC cancer 2021;21:542.

54. Qu F, Li Z, Lai S, et al. Construction and Validation of a Serum Albumin-to-Alkaline Phosphatase Ratio-Based Nomogram for Predicting Pathological Complete Response in Breast Cancer. Frontiers in Oncology 2021;11.

55. Jiang C, Xiu Y, Yu X, Qiao K, Zhang S, Huang Y. Prognostic value of a modified systemic inflammation score in breast cancer patients who underwent neoadjuvant chemotherapy. BMC Cancer 2022;22.

56. Qiong Z, Jie D, Qingqing S, Nannan L, Yueyin P, Xinghua H. Role of neutrophil-to-lymphocyte ratio as a prognostic biomarker in patients with breast cancer receiving neoadjuvant chemotherapy: a meta-analysis. BMJ Open 2021;11:e047957.

57. Hamid HKS, Davis GN, Trejo-Avila M, Igwe PO, Garcia-Marín A. Prognostic and predictive value of neutrophil-to-lymphocyte ratio after curative rectal cancer resection: A systematic review and meta-analysis. Surg Oncol 2021;37:101556.

58. Zhu Y, Si W, Sun Q, Qin B, Zhao W, Yang J. Platelet-lymphocyte ratio acts as an indicator of poor prognosis in patients with breast cancer. Oncotarget 2017;8:1023-30.

59. Zhang M, Huang XZ, Song YX, Gao P, Sun JX, Wang ZN. High Platelet-to-Lymphocyte Ratio Predicts Poor Prognosis and Clinicopathological Characteristics in Patients with Breast Cancer: A Meta-Analysis. Biomed Res Int 2017;2017:9503025.

60. Gong Z, Xin R, Li L, Lv L, Wu X. Platelet-to-lymphocyte ratio associated with the clinicopathological features and prognostic value of breast cancer: A meta-analysis. The International Journal of Biological Markers 2022;37:339-48.

61. Long Y, Zhang Y, Ni L, et al. Prognostic value of platelet-to-lymphocyte ratio in neoadjuvant chemotherapy for solid tumors: A PRISMA-compliant meta-analysis. Medicine (Baltimore) 2021;100:e26202.

62. Li X, Lin H, Ouyang R, Yang Y, Peng J. Prognostic significance of the systemic immune-inflammation index in pancreatic carcinoma patients: a meta-analysis. Biosci Rep 2021;41.

63. Ji Y, Wang H. Prognostic prediction of systemic immune-inflammation index for patients with gynecological and breast cancers: a meta-analysis. World J Surg Oncol 2020;18:197.

64. Wang B, Huang Y, Lin T. Prognostic impact of elevated pre-treatment systemic immune-inflammation index (SII) in hepatocellular carcinoma: A meta-analysis. Medicine (Baltimore) 2020;99:e18571.

65. Wei L, Xie H, Yan P. Prognostic value of the systemic inflammation response index in human malignancy: A meta-analysis. Medicine (Baltimore) 2020;99:e23486.

66. Hu RJ, Liu Q, Ma JY, Zhou J, Liu G. Preoperative lymphocyte-to-monocyte ratio predicts breast cancer outcome: A meta-analysis. Clin Chim Acta 2018;484:1-6.

67. Yang XC, Liu H, Liu DC, Tong C, Liang XW, Chen RH. Prognostic value of pan-immune-inflammation value in colorectal cancer patients: A systematic review and meta-analysis. Front Oncol 2022;12:1036890.

68. Kuerer HM, Smith BD, Krishnamurthy S, et al. Eliminating breast surgery for invasive breast cancer in exceptional responders to neoadjuvant systemic therapy: a multicentre, single-arm, phase 2 trial. Lancet Oncol 2022;23:1517-24.

69. Pfob A, Sidey-Gibbons C, Rauch G, et al. Intelligent Vacuum-Assisted Biopsy to Identify Breast Cancer Patients With Pathologic Complete Response (ypT0 and ypN0) After Neoadjuvant Systemic Treatment for Omission of Breast and Axillary Surgery. J Clin Oncol 2022;40:1903-15.

70. Jung J-J, Kim S-Y, Koh J, et al. Omission of breast surgery for predicted pCR patients with MRI and vacuum-assisted biopsy in breast cancer after neoadjuvant chemotherapy: A multicenter, single-arm, non-inferiority trial (OPTIMIST trial). Journal of Clinical Oncology 2023;41:TPS627-TPS.

71. Hu Y, Wang S, Ding N, Li N, Huang J, Xiao Z. Platelet/Lymphocyte Ratio Is Superior to Neutrophil/Lymphocyte Ratio as a Predictor of Chemotherapy Response and Disease-free Survival in Luminal B-like (HER2−) Breast Cancer. Clinical Breast Cancer 2020;20:e403-e9.

72. Jiang C, Zhang S, Qiao K, Xiu Y, Yu X, Huang Y. The Pretreatment Systemic Inflammation Response Index as a Useful Prognostic Factor is Better Than Lymphocyte to Monocyte Ratio in Breast Cancer Patients Receiving Neoadjuvant Chemotherapy. Clinical Breast Cancer 2022;22:424-38.

73. Y L, J Z, B W, H Z, J H, K W. Development and Validation of a Nomogram to Predict the Probability of Breast Cancer Pathologic Complete Response after Neoadjuvant Chemotherapy: A Retrospective Cohort Study. Frontiers in surgery 2022;9:878255.

74. Lou C, Jin F, Zhao Q, Qi H. Correlation of serum NLR, PLR and HALP with efficacy of neoadjuvant chemotherapy and prognosis of triple-negative breast cancer. American Journal of Translational Research 2022;14:3240-6.

75. M Z, L C, X K, et al. The Systemic Immune-Inflammation Index is an Independent Predictor of Survival in Breast Cancer Patients. Cancer management and research 2022;14:775-820.

76. M Z, L C, X K, et al. The Systemic Inflammation Response Index as an Independent Predictor of Survival in Breast Cancer Patients: A Retrospective Study. Frontiers in molecular biosciences 2022;9:856064.

77. Chen L, Kong X, Wang Z, Wang X, Fang Y, Wang J. Pretreatment systemic inflammation response index in patients with breast cancer treated with neoadjuvant chemotherapy as a useful prognostic indicator. Cancer Management and Research 2020;12:1543-67.
